# Similar incidence and implant survival of multiply revised knee arthroplasties in Norway and Denmark: a register study of the Danish and Norwegian Knee Arthroplasty registries from 1998–2021

**DOI:** 10.2340/17453674.2026.45411

**Published:** 2026-02-20

**Authors:** Julius Tetens HALD, Anne Marie FENSTAD, Anders EL-GALALY, Anders ODGAARD, Ove Nord FURNES

**Affiliations:** 1 Department of Orthopaedic Surgery, Rigshospitalet, Copenhagen University, Denmark; 2 The Norwegian Arthroplasty Register, Department of Orthopedic Surgery, Haukeland University Hospital, Bergen, Norway; 3 Department of Clinical Medicine, University of Copenhagen, Copenhagen, Denmark; 4 Department of Clinical Medicine, Faculty of Medicine, University of Bergen, Bergen, Norway

## Abstract

**Background and purpose:**

The aim of our study was to identify the absolute incidence and implant survival probability of multiply revised knee arthroplasties in Norway and Denmark from 1998 to 2021.

**Methods:**

This was an observational study of primary and revision knee arthroplasties reported prospectively in Norway and Denmark. The nationwide registers in Norway and Denmark were utilized. After identification of all primary procedures reported from 1998 to 2021, revision procedures were identified. Probabilities of implant survival were calculated using Kaplan–Meier methods. A multiply revised knee arthroplasty was defined as at least 3 revisions of a primary knee arthroplasty.

**Results:**

The proportion of third revisions was 0.4% in both Denmark (621/159,343) and Norway (404/105,192). The 16-year implant survival of third revisions was 57% (CI 52–62) in Denmark and 54% (CI 48–61) in Norway. No difference in the risk of revision between countries was found for the third revision (HR 1.1, CI 0.7–1.8).

**Conclusion:**

The incidence of multiply revised knee arthroplasties was not significantly different between Norway and Denmark. The risk and implant survival probability of multiply revised knee arthroplasties was similar in the 2 countries. The results can be used as a benchmark for incidence and prevalence calculations in other countries.

The overall risk of experiencing three or more revisions of a primary knee arthroplasty is generally considered to be low [1]. However, it has been demonstrated that each subsequent revision following the primary knee arthroplasty has a significant negative impact on implant survival [1,2]. This is supported by 2 studies conducted in the United Kingdom and Denmark [1,2]. The study from the UK analyzed over 1 million primary knee arthroplasties and more than 33,000 first revisions over a 15-year period. The study then identified 3,500 second revisions and more than 500 third revisions coupled to the primary arthroplasty. The Danish study found that the proportion of multiply revised knee arthroplasties increased from 2010 to 2021 compared with 1998 to 2009. Both studies reported that relatively few patients underwent a third revision, but the 10-year implant survival of third revisions ranged from 60% to 70%.

Given the rising number of primary knee arthroplasties and the increasing proportion of younger patients with a higher lifetime risk of revision, national populations can generally expect a growing number of multiply revised knee arthroplasties [3]. However, no study has systematically compared the incidence and implant survival of these procedures across 2 national populations. Understanding such differences may help identify factors influencing revision patterns. The aim of this study was to identify the absolute incidence and implant survival probability of multiple knee arthroplasty revisions in Norway and Denmark from 1998 to 2021.

## Methods

### Design and data sources

This was an observational register study and adheres to the STROBE guidelines [4]. The study used 2 national databases from Denmark and 1 national database from Norway: the Danish National Patient Registry (DNPR), the Danish Knee Arthroplasty Register (DKAR), and the Norwegian Arthroplasty Register (NAR). The DNPR is a national administrative database for the Danish healthcare system [5]. The DKAR is a clinical quality database containing clinical and technical information on knee arthroplasties in Denmark [6]. The DKAR covered 96.6% of all primary procedures and 96.1% of revision procedures in Denmark in 2021. However, the completeness of the register was low at the beginning of the observation period. For this reason, data from DNPR was included. The NAR is also a clinical quality register containing data on knee arthroplasties in Norway [7,8]. The NAR covered 96.5% of all primary procedures and 93% of revision procedures in Norway in 2019/2020 [9]. The completeness of NAR has been excellent throughout the observation period. The registers were searched for information on all primary and revision knee arthroplasties. Information regarding death and emigration were collected through the National Population Registry in Norway (skatteetaten.no), and the Danish Civil Registration System (DCRS) [10]. All citizens and permanent residents in Norway and Denmark receive a unique identifier, which follows all individuals from birth or immigration until death or emigration. Both countries have public and tax-funded healthcare.

### Study population and definitions

The registers were queried for all primary knee arthroplasty procedures reported from January 1, 1998 to December 31, 2021 in Denmark and Norway. The unit of observation was knees, and patients could contribute with both knees. For Denmark, both the DNPR and DKAR were searched for primary procedures and then revisions. From the primary knee population, revision procedures were identified. Death and emigration resulted in censoring. Thus, the sequence of revisions was established. Eligibility was defined as any primary knee arthroplasty from January 1, 1998 to December 31, 2021 on a person resident in Norway and Denmark at the time of the procedure. All types of implants were eligible, such as total knee arthroplasty (TKA) and unicompartmental knee arthroplasty (UKA). Revision was defined as any surgical procedure with the exchange, removal, or addition of an arthroplasty component, thus 2-stage revisions were counted as 2 procedures [11]. However, knee arthrodesis and amputation were not included in this study. A multiply revised knee arthroplasty was defined as a primary knee arthroplasty which subsequently experienced at least 3 revisions.

### Outcomes and statistics

The primary outcome of this study was the absolute incidence and implant survival probability of multiple knee arthroplasty revisions in Norway and Denmark from January 1, 1998, to December 31, 2021. The Kaplan–Meier method was used to calculate the implant survival of each procedure grouped by the country of origin, to report the absolute incidence and to account for differences in follow-up and censoring. Follow-up was censored at the date of death or emigration. Revision was defined as the event of interest. Patients were followed until December 31, 2021. Cox regression, adjusted for age, sex, and whether the primary procedure was performed before or after January 1, 2010, was used to compare differences in revision risk between the 2 countries. The observation periods were divided when performing Cox regression to accommodate the proportional hazards assumption. These divisions were based on visual inspection of the survival curves and on Schoenfeld residuals plots. Tables were used to describe baseline variables, results and include missing data. Standardized mean difference (SMD) and two-proportion z-test were used to show differences between groups. RStudio version 4.2 was used for all calculations (R Foundation for Statistical Computing, Vienna, Austria).

### Approvals, ethics, data sharing, funding, and disclosures

The Capital Region gave approval for data storage and analysis. This was given on behalf of the Danish Data Protection Agency (case number: P-2022-711). Access to data is available from Statistics Denmark, the DKAR, and the NAR, and required approval from these institutions. The study was funded by Rigshospitalets Forskningspulje, sundhedsdonationer.dk, Copenhagen University, Aase and Ejnar Danielsens Fond, William Demant Fonden, Gehejmeråd P. Hersleb Classens Rejsestipendium for Læger, and Carl and Ellen Hertz’s Science Grant. The Norwegian Arthroplasty Register has permission from the Norwegian Data Inspectorate to collect patient data based on written consent from the patient (ref 24.1.2021: 16/01622-3/CDG). No conflicts of interest were declared. Complete disclosure of interest forms according to ICMJE are available on the article page, doi: 10.2340/17453674.2026.45411

## Results

**Figure 1:**
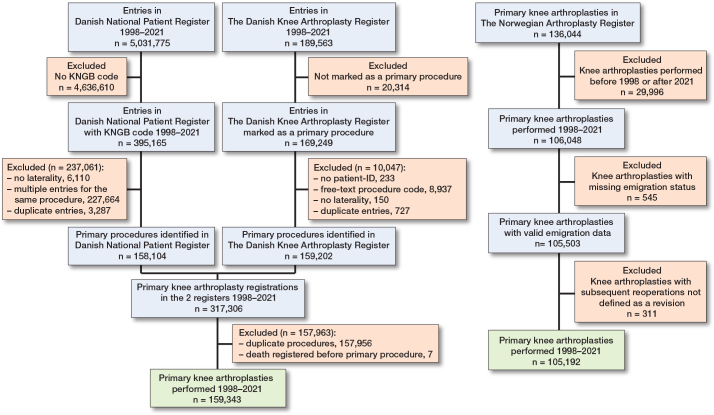
Flowchart of patient inclusion process in Denmark and Norway.

After querying the registers from the 2 countries, we identified 159,343 primary knee arthroplasties in Denmark and 105,192 primary knee arthroplasties in Norway (Figure 1). The median age and proportion of females were statistically significantly different, but the differences were small (SMD –0.1 for age, SMD 0.03 for sex) (Table 1). In both countries, the most common implant was TKA, with similar proportions of UKA and patellofemoral arthroplasty (PFA). The largest difference was observed in the use of a patellar component: 21% (28,940/135,550) of TKAs in Denmark were reported without a patellar component, compared with 93% (85,406/91,727) in Norway. The distribution of cemented and cementless implants was relatively similar, though cementless implants were more commonly used in Denmark. The indications for the primary procedure were also similar in the 2 countries.

**Table 1:** Patient characteristics

Factor	Denmark n = 159,343	Norway n = 105,192	SMD [CI]
Age, median (IQR)	68 (61–75)	69 (62–76)	–0.1 [–0.1 to –0.1]
Female sex	94,864 (60)	64,153 (61)	0.03 [0.02 to 0.04]
Institution			0.4 [0.4 to 0.5]
Public	133,139 (84)	101,528 (97)	
Private	26,154 (16)	3,664 (3.5)	
Unknown	50 (< 0.1)	0 (0)	
Implant			0.2 [0.2 to 0.2]
TKA	135,550 (85)	91,727 (87)	
TKA with patellar comp.	99,053/135,550 (73)	6,321/91,727 (6.9)	
Cemented patella	98,267/99,053 (99)	5,764/6,321 (91)	
Uncemented patella	604/99,053 (0.6)	189/6,321 (3.0)	
Unknown fixation	182/99,053 (0.2)	368/6,321 (5.8)	
TKA without patellar comp.	28,940/135,550 (21)	85,406/91,727 (93)	
TKA, unknown patellar comp.	7,557/135,550 (5.6)	0/91,727 (0)	
Unicondylar arthroplasty **^a^**	18,554 (12)	12,803 (12)	
Patellofemoral arthroplasty	1,885 (1.2)	650 (0.6)	
Unknown arthroplasty	3,354 (2.1)	12 (<0.1)	
Fixation of tibial comp.			0.3 [0.3 to 0.3]
Cemented	127,699 (80)	93,740 (89)	
Cementless	21,114 (13)	10,617 (10)	
Unknown	10,530 (6.6)	835 (0.8)	
Fixation of femoral comp.			0.4 [0.4 to 0.4]
Cemented	103,669 (65)	82,161 (78)	
Cementless	46,818 (29)	22,857 (22)	
Unknown	8,856 (5.6)	174 (0.2)	
Indication for procedure			0.4 [0.4 to 0.4]
Primary osteoarthrosis	124,632 (78)	88,786 (84)	
Secondary osteoarthrosis **^b^**	18,588 (12)	10,929 (10)	
Arthritis **^c^**	3,997 (2.5)	3,719 (3.5)	
Other **^d^**	1,288 (0.8)	1,621 (1.5)	
Unknown	10,838 (6.8)	137 (0.1)	

Comp.: component, IQR = 25% to 75% percentiles, SMD = standardized mean difference, TKA = total knee arthroplasty.

a Unicondylar prostheses included medial and lateral unicompartmental knee arthroplasties.

b Secondary arthrosis included sequelae after fractures, ligament and meniscus injuries.

c Arthritis included rheumatoid arthritis and sequelae after other arthritides such as Mb. Bechterew.

d Other indications included tumors, osteonecrosis, and other rare indications.

In Denmark, we identified 10,544 first revisions, 2,129 second revisions, 621 third revisions, and 222 fourth revisions (Table 2). In Norway, we identified 5,980 first revisions, 1,299 second revisions, 404 third revisions, and 157 fourth revisions. A statistically significant difference was found for the first and second revisions concerning the relative proportions of revisions to primary procedures with higher proportions found for Denmark (Table 2). The distribution of the indication of the first revisions is presented in Table 3. The “big three” indications in the merged data set were infection, loosening, and instability. In Denmark 1,940/10,544 (18%) of the revisions were for infections, whereas in Norway this was 1,171/5,980 (20%). In Denmark, 3,881/10,544 (37%) of the revisions were due to loosening, wear, osteolysis or fractures. In Norway, this was 1,722/5,980 (29%) of the revisions. There were nearly similar percentages of revisions due to instability, malalignment, and pain only.

**Table 2:** Incidence of revisions. Values are count (%)

Revision	Denmark n = 159,343 ^a^	Norway n = 105,192 ^a^	Difference ^b^ (CI)	P value
First	10,544 (6.6)	5,980 (5.7)	0.9% (0.7 to 1.1)	< 0.001
Second	2,129 (1.3)	1,299 (1.2)	0.1% (0.01 to 0.2)	0.03
Third	621 (0.4)	404 (0.4)	0.0% (–0.04 to 0.05)	0.8
Fourth	222 (0.1)	157 (0.1)	0.0% (–0.04 to 0.02)	0.5

**Table 3:** Indications for revisions. Values are count (row wise %)

Factor	Total n	Infection	Loosening,wear, osteolysis, fracture	Instability, malalignment	Progression of arthrosis	Other	Pain	Missing	SMD (CI) ^a^
First revisions									0.2 (0.2–0.3)
Denmark	10,544	1,940 (18)	3,881 (37)	1,809 (17)	914 (8.7)	811 (7.7)	1,118 (11)	71 (0.7)	
Norway	5,980	1,171 (20)	1,722 (29)	1,379 (23)	450 (7.5)	410 (6.9)	823 (14)	25 (0.4)	
Second revisions									0.3 (0.2–0.4)
Denmark	2,129	785 (37)	575 (27)	416 (20)	37 (1.7)	139 (6.5)	130 (6.1)	47 (2.2)	
Norway	1,299	505 (39)	238 (18)	277 (21)	25 (1.9)	153 (12)	98 (7.5)	3 (0.2)	
Third revisions									0.4 (0.3–0.5)
Denmark	621	315 (51)	111 (18)	107 (17)	8 (1.3)	32 (5.2)	32 (5.2)	16 (2.6)	
Norway	404	210 (52)	57 (14)	51 (13)	3 (0.7)	62 (15)	21 (5.2)	0 (0)	
Fourth revisions									0.6 (0.4–0.8)
Denmark	222	133 (60)	36 (16)	33 (15)	0 (0)	9 (4.1)	6 (2.7)	5 (2.3)	
Norway	157	88 (56)	18 (11)	10 (6.4)	0 (0)	31 (20)	10 (6.4)	0 (0)	

a Standardized mean difference (95% confidence interval).

### Implant survival of primary arthroplasties

**Figure 2:**
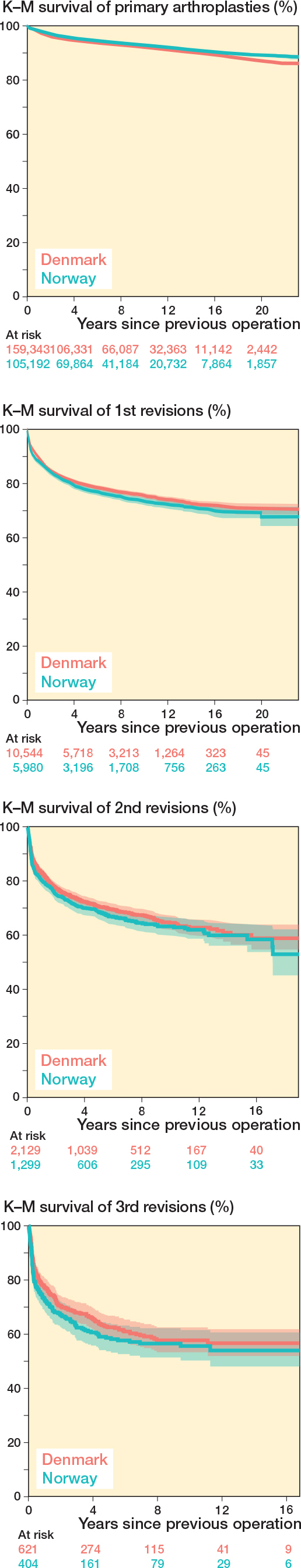
Survival probability of multiple revisions grouped by country. New risk sets are created after each event with follow-up reset after each revision.

The 16-year implant survival in Denmark was 89% (95% confidence interval [CI] 89–90) (Figure 2). In Norway, this was 90% (CI 90–91). In the first 2 years, the risk of a primary revision adjusted for age, sex, and time-period was lower (HR 0.8, CI 0.8–0.9) for Norway compared with Denmark. From year 2 to 16, the risk of revision adjusted for age, sex, and time-period was higher (HR 1.2, CI 1.1–1.2) for Norway compared with Denmark. From year 16 to 22, the risk of revision adjusted for age, sex, and time-period was lower (HR 0.6, CI 0.4–0.8) for Norway than for Denmark.

### Implant survival of first revisions

The 16-year implant survival in Denmark was 72% (CI 71–74) (see Figure 2). In Norway, this was 70% (CI 68–72). In the first 2 years, the risk of a second revision adjusted for age, sex, and time-period was higher (HR 1.1, CI 1.01–1.2) in Norway compared with Denmark. From year 2 to 16, the risk of revision adjusted for age, sex, and time-period was higher (HR 1.3, CI 1.1–1.5) in Norway than in Denmark. From year 16 to 22, the risk of revision adjusted for age, sex, and time-period was similar (HR 0.8, CI 0.2–3.4) in Norway than in Denmark.

### Implant survival of second revisions

The 16-year implant survival in Denmark was 59% (CI 55–64) (see Figure 2). In Norway, this was also 59% (CI 54–64). In the first 2 years, the risk of a third revision adjusted for age, sex, and time-period was similar (HR 1.0, CI 0.9–1.2) in Norway compared with Denmark. Also from year 2 to 18, the risk of revision adjusted for age, sex, and time-period was similar (HR 1.1, CI 0.8–1.4) in Norway compared with Denmark.

### Implant survival of third revisions

The 16-year implant survival in Denmark was 57% (CI 52–62) (see Figure 2). In Norway, this was 54% (CI 48–61). In the first 2 years, the risk of a fourth revision adjusted for age, sex, and time-period was similar (HR 1.2, CI 0.9–1.5) in Norway compared with Denmark. Also from year 2 to 16, the risk of revision was similar (HR 1.1, CI 0.7–1.8) in Norway compared with Denmark.

### TKA and UKA

**Figure 3:**
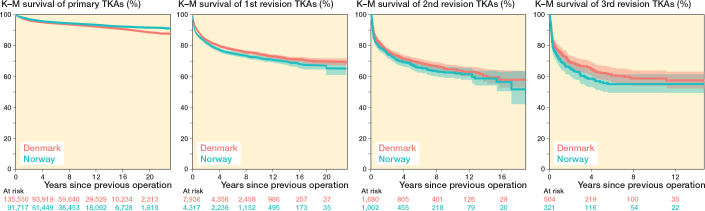
Survival probability of multiple revisions for primary TKAs grouped by country.

**Figure 4:**
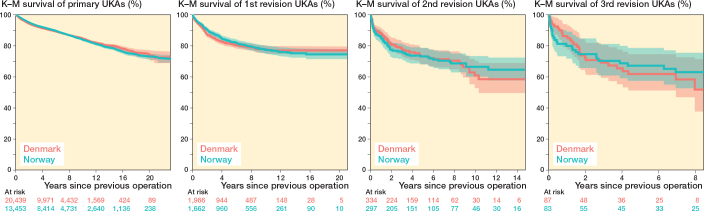
Survival probability of multiple revisions for primary UKAs grouped by country.

The incidences of multiple revision for primary TKAs and UKAs showed that primary UKAs in Norway demonstrated a higher proportion of knees requiring multiple subsequent revisions than Denmark (–2.8% in Denmark compared with Norway) (Tables 4 and 5). The implant survival probabilities of knees with a primary TKA are shown in Figure 3. The implant survival probabilities of knees with a primary UKA are shown in Figure 4.

**Table 4:** Incidence of revisions for primary total knee arthroplasties (TKAs). Values are count (%)

Revision	Denmark n = 135,550 ^a^	Norway n = 91,727 ^a^	Difference ^b^ (CI)	P value
First	7,936 (5.9)	4,317 (4.7)	1.2% (1.0 to 1.3)	<0.001
Second	1,680 (1.2)	1,002 (1.1)	0.1% (0.06 to 0.2)	0.001
Third	504 (0.4)	321 (0.3)	0.1% (–0.03 to 0.07)	0.4
Fourth	182 (0.1)	128 (0.1)	0.0% (–0.04 to 0.03)	0.8

a Primary TKAs,

b Two-proportion z-test.

**Table 5:** Incidence of revisions for primary unicompartmental knee arthroplasties (UKAs) and patellofemoral arthroplasties (PFAs). Values are count (%)

Revision	Denmark n = 20,439 ^a^	Norway n = 13,453 ^a^	Difference ^b^ (CI)	P value
First	1,966 (9.6)	1,662 (12.4)	–2.8% (–3.4 to –2.0)	<0.001
Second	334 (1.6)	297 (2.2)	–0.6% (–0.9 to –0.2)	<0.001
Third	87 (0.4)	83 (0.6)	–0.2% (–0.4 to –0.03)	0.02
Fourth	29 (0.1)	29 (0.2)	–0.1% (–0.2 to 0.1)	0.1

a Primary UKAs and PFAs,

b Two-proportion z-test.

## Discussion

The aim of our study was to identify the absolute incidence and implant survival probability of multiply revised knee arthroplasties in Norway and Denmark from 1998 to 2021.

We found that the incidence of multiply revised knee arthroplasties relative to the number of primary arthroplasties were similar between the 2 countries. This proportion was 0.4% (621/159,373) in Denmark and 0.4% (404/105,192) in Norway. Also, this study found that the implant survival probabilities of multiply revised knee arthroplasties were similar across the 2 countries. The 16-year implant survival of third revisions was 57% (95% CI 52–62%) in Denmark and 54% [95% CI 48–61%] in Norway. No difference in the risk of revision was found for the implant survival of the second and third revisions. Overall, there were small differences between the 2 countries.

Our findings agree with previous registry-based studies from the United Kingdom and Denmark [1,2]. These studies showed that the survival of second and third revisions is considerably lower than that of primary procedures and first revision procedures, with 10-year implant survival rates ranging from 60% to 70%. We found similar probabilities of implant survival in Norway. Thus, the argument that implant survival of knee arthroplasties decreases after each subsequent revision appears strengthened. There may possibly be a gradual diminishment of survival differences as the ordinal number of a revision increases. In our data, the nominal difference in survival from primary to first revision was 19% (from 90% to 71%), the nominal difference from first to second revision was about 12% (from 71% to 59%), while the nominal difference from second to third revision was only 3% (from 59% to 56%). This may suggest a reduced inclination among patients and surgeons to proceed with another revision, in addition to some patients receiving end-stage treatment, such as knee arthrodesis or femoral amputation.

One of the most striking differences between Norway and Denmark is the use of a patellar component at the time of primary arthroplasty. The proportion of TKAs with a patellar component was much higher in Denmark than in Norway. The impact of this difference on revision rates and implant survival is unclear. A study using data from the NAR found that the overall risk of revision for resurfaced TKAs were marginally lower than for unsurfaced TKAs but that resurfaced TKAs had higher risks of revision for some stratified indications [12] and there were no differences in pain and function [13]. A systematic review of overlapping meta-analyses found no clear superiority of resurfaced TKAs compared with unsurfaced TKAs [14].

We found that, relative to the number of primary procedures, UKAs in Norway underwent multiple revisions more frequently than in Denmark. In Denmark, the proportion was similarly high for the first 2 revisions when compared with the proportion of all implants. Registry studies have previously demonstrated that UKAs have a higher revision rate than TKAs [15]. However, this does not necessarily indicate that UKAs are inferior implants; rather, revision rates may be influenced by the surgeon’s threshold for revision, and UKAs have been shown to be revised with higher Oxford Knee Scores (OKS) and better range of motion (ROM) than TKAs [16]. Additionally, surgeon experience tends to vary more for UKAs than for TKAs, as UKAs are reported less frequently by some surgeons [17]. Hypothetically speaking, if UKA procedures in Denmark were more centralized in specialized centers, while in Norway their use was more dependent on individual surgeon preference, this could contribute to inter-country differences. Variations in revision thresholds and surgeon experience in performing UKAs between Denmark and Norway may therefore explain part of the differences observed in our study [18].

### Strengths

The strength of this study lies in its utilization of national arthroplasty registers from 2 countries with high-quality data collection. The inclusion of 3 independent registries (DNPR, DKAR, NAR) enhances the generalizability of the findings and provides robust estimates of revision rates and implant survival.

### Limitations

Our results rely on accurate reporting to the registers; mistakes in these may therefore bias our results. The number of multiple revisions performed is small, even with large cohorts of primary knee arthroplasties. This affects the statistical power of our analysis. One limitation is the underreporting of infections in the DKAR [19]. In addition, due to low data coverage at the beginning of the observation period, we included diagnosis codes from the DNPR to identify indications when this information was missing from the DKAR. This was not done in the Norwegian population due to the excellent coverage in NAR. The accuracy of identifying infections in NAR is not known, which may influence analyses of infections. Additionally, while both national registries have high coverage, the completeness of covariates like indication for revision and type of implant fixation differed between the 2 countries, which could influence some comparisons.

### Conclusion

We showed that the incidence of multiply revised knee arthroplasties was similar in Norway and Denmark. We found that the implant survival of multiply revised knee arthroplasty patients decreased for each subsequent revision by similar amounts across 2 national populations in Denmark and Norway.

*In perspective*, our study highlights the clinical difficulties associated with multiply revised knee arthroplasty patients who have poor implant survival after the first revision.

## Notes

Handling co-editors: Keijo Mäkelä and Philippe Wagner

*Acta* thanks Mika Niemeläinen and Annette W-Dahl for help with peer review of this manuscript.
